# Supplementary material for: Hybrid Models and Biological Model Reduction with PyDSTool
Source: PLoS Comput Biol. 2012 Aug 9;8(8):e1002628. doi: 10.1371/journal.pcbi.1002628 (PMC3415397; doi:10.1371/journal.pcbi.1002628)
Supplement: Text S4 — Complete source code for the PyDSTool package (version 0.88.120504). Includes API documentation and help files linking to web pages. This file is identical to the current public release on Sourceforge.net. (ZIP) [file pcbi.1002628.s004.zip › PyDSTool/html/PyDSTool.Toolbox.optimizers.step.local_brute_force_1dstep-module.html]

xml version="1.0" encoding="ascii"?


PyDSTool.Toolbox.optimizers.step.local\_brute\_force\_1dstep


| Home | Trees | Indices | Help | PyDSTool |
| --- | --- | --- | --- | --- |

Package PyDSTool ::
Package Toolbox ::
Package optimizers ::
Package step ::
Module local\_brute\_force\_1dstep

[hide private]

[frames] | no frames]

# Module local\_brute\_force\_1dstep

source code


|  |  |
| --- | --- |
| Classes | [hide private] |

LocalBruteForce1DStep  
Local brute force search for 1D parameter (sub-)space, making no
use of gradient information.

| Home | Trees | Indices | Help | PyDSTool |
| --- | --- | --- | --- | --- |

|  |  |
| --- | --- |
| Generated by Epydoc 3.0.1 on Fri May 4 15:24:05 2012 | http://epydoc.sourceforge.net |
